# Evaluating Experiences With the Newly Enacted Law on Assisted Suicide in Austria: Protocol for an Interdisciplinary Mixed Methods Study

**DOI:** 10.2196/86740

**Published:** 2026-04-16

**Authors:** Klara Doppler, Tamina-Laetitia Vielgrader, Julia Fischer, Helene Mayer, Jana Marica Hluch, Marlene Walser, Sabine Parrag, Stefan Dinges, Karl Stöger, Thomas Wochele-Thoma, Maria Kletecka-Pulker

**Affiliations:** 1 Department for Ethics and Law in Medicine, University of Vienna/Medical University of Vienna, Spitalgasse 2-4, Hof 2.8, Vienna, 1090, Austria; 2 Department of Constitutional and Administrative Law, Faculty of Law, University of Vienna, Vienna, Austria; 3 Ludwig Boltzmann Gesellschaft, Ludwig Boltzmann Institute Digital Health and Patient Safety, Vienna, Austria

**Keywords:** assisted suicide, assisted dying, end of life, interdisciplinary studies, empirical research, legal aspects, medical ethics, social sciences, stakeholder participation

## Abstract

**Background:**

Austria has only recently established a legal framework for assisted dying. Individuals seeking assistance in suicide must navigate a multistage process that has been criticized for its complexity both for the individuals seeking assistance and for the health care and legal professionals involved in the procedure.

**Objective:**

To address the critical gap in empirical research that sheds light on the law’s implementation and its impact on the lived experiences of those involved, as well as on the broader health care sector, we have designed an evaluation that examines the various perspectives of individuals seeking assisted suicide; their family members and other relatives; and all professionals specified in the legal framework—notably physicians, notaries and other relevant legal professionals, pharmacists, and psychologists.

**Methods:**

Our study uses an interdisciplinary approach that integrates theories, concepts, and methodologies from legal science, ethics, and social science. The legal and ethical components examine aspects of the Austrian legal framework, such as the legal foundations, moral implications, and ethical challenges involved in assisted dying. The main part of this study lies in its comprehensive social science section, which consists of 2 online cross-sectional surveys. The surveys assess sociodemographic characteristics, knowledge and understanding of the framework, encounters and experiences with assisted suicide, and reported need for support and information on the legalization of assisted suicide. In parallel, we are in the process of conducting semistructured in-depth interviews with (1) individuals seeking assistance in dying, (2) family members and other relatives of the individuals seeking assistance in dying, and (3) professionals mentioned in the legal framework (eg, physicians). Thematic analysis will be used to interpret the interview data.

**Results:**

The initial research proposal received approval from the University of Vienna Ethics Committee in November 2023. The legal and ethical analyses are ongoing. Recruitment for interview participants began in January 2024. In total, 49 participants have been interviewed as of February 2026. Given the novelty of the legislation and the limited practical experience to date, further interviews are scheduled until April 2026. Data collection for both surveys took place from June to October 2024. The final samples consisted of 239 medical and nursing directors and 304 physicians. Data analysis was completed in May 2025. Completion and dissemination of all study components are anticipated by the end of 2026.

**Conclusions:**

Our multiperspective evaluation aims to assess the framework on assisted suicide in Austria. By evaluating the perspectives of relevant key stakeholders, we aim to provide a nuanced understanding of the law’s societal, legal, and ethical implications.

## Introduction

### Background

In January 2022, Austria established a legal framework for assisted suicide, making it one of the most recent countries to regulate assisted dying amid intensifying global debate and a growing number of nations either adopting or considering relevant legal frameworks [1-7].

The political debate on self-determined dying and assisted suicide in Austria had been underway for many years, increasing as more European countries introduced legal frameworks. However, apart from a 2015 statement by the Austrian Bioethics Commission advocating reform [8], no significant political steps were taken until the Austrian Constitutional Court’s 2020 ruling, which declared the outright prohibition of assisted suicide to be unconstitutional [9], ultimately leading to the introduction of the Sterbeverfügungsgesetz (Dying Decree Act) on January 1, 2022. The law supplements the Austrian Criminal Code and introduces the concept of the “dying decree” (*Sterbeverfügung*). A dying decree is a formal written declaration in which an individual who has decided to pursue assisted suicide records their voluntary and autonomous decision to do so. Health care and legal professionals are involved in preparing a dying decree, which permits the substance pentobarbital to be dispensed by a public pharmacy.

The eligibility criteria for obtaining assistance in suicide are shaped by a restrictive legal framework, ultimately limiting access to this option to a small group of individuals. To qualify, individuals must be of legal age and fully capable of making informed decisions. Additionally, the option is only available to individuals who are either Austrian citizens or who have permanent residence in Austria.

Moreover, the law enforces strict conditions with respect to the individual’s state of health: a dying decree may only be issued to people with either an incurable terminal illness or a severe, permanent illness with persistent symptoms that permanently impair their overall quality of life. In both cases, the illness must result in a subjectively unbearable level of suffering that cannot otherwise be alleviated.

Beyond these strict personal requirements, individuals who wish to terminate their lives must navigate significant hurdles during the subsequent procedural steps of preparing a dying decree. The formal process begins with a medical assessment by 2 physicians, one of whom must be certified in the field of palliative medicine. If there is an indication that the individual has a psychological condition that could prompt the desire to end their life, this must additionally be assessed by a psychiatric specialist or clinical psychologist.

After a 12-week waiting period (shortened to 2 weeks if the terminal phase has already been reached), the next step is for a notary or legally qualified employee of a public patient advocacy institution to formally establish or issue the decree. The process is then finalized by providing the individual who wishes to terminate their life via dying decree with a lethal preparation of pentobarbital that can be dispensed by a public pharmacy (Textbox 1).

Textbox 1. **Dying Decree Act, Austria (2022).**

**Eligibility criteria**

- Permanent residence in Austria or Austrian citizenship
- At least 18 years of age
- Full capability of making an informed decision
- An incurable terminal illness or a severe, permanent illness with persistent symptoms, the effects of which permanently impair the person’s entire lifestyle; the illness must result in a level of suffering that cannot otherwise be alleviated

**Procedural requirements**

- Two independent eligibility assessments by physicians, one of whom must be certified in the field of palliative medicine
- The physicians must inform the individual seeking assisted suicide about (1) alternative courses of action; (2) the availability of psychotherapeutic and other counseling services; and (3) the dosage, accompanying medication, and method of administration of the lethal preparation
- Optional third assessment by a psychiatric specialist or clinical psychologist in cases in which there is an indication that the individual seeking assisted suicide has a psychological condition
- Waiting period of 12 weeks between the first medical consultation and the effective issue of a dying decree; this period can be shortened to 2 weeks if the illness has already progressed to the terminal phase
- The decree is formally issued by a notary or a legally qualified employee of a public patient advocacy institution
- Proof of the valid dying decree must be presented when the preparation is dispensed by the pharmacy

In terms of the final steps of assisted suicide, the law provides no further procedural guidelines. In Austria, assisted suicide is not institutionalized, nor are physicians or other health care providers obligated to accompany individuals through the last step. The legislator has intentionally left the final steps of assisted suicide largely open-ended. In practice, this lack of guidance has resulted in hurdles for all parties involved.

While the Austrian legal framework is broadly in line with international practices [1-7], the regulatory approach toward the final step of the assisted suicide process is comparatively atypical. In most other jurisdictions—with the exception of Germany (which lacks a specific statutory framework) and Switzerland (where the law does not formally mandate such involvement, although, in practice, a highly structured process ensures professional participation)—medical professionals hold clearly defined responsibilities regarding the application of the lethal preparation [1]. In contrast, the Austrian model leaves the application of the barbiturate unsupervised, lacking a formal requirement for health care professionals to accompany the individual at the time of the assisted suicide.

The unique challenges posed by the Dying Decree Act in Austria have been the subject of theoretical discourse, particularly in the legal [10-13] and ethical [14-16] realms. The law was highly controversial and widely debated, which eventually brought it to the Austrian Constitutional Court for examination. In December 2024, the Constitutional Court confirmed that most provisions of the Dying Decree Act conformed to the Federal Constitution. Two provisions (the need for full renewal of a dying decree after 1 year and the strict ban on information about relevant services) were declared unconstitutional.

However, 3 years after the law’s implementation, by early 2025, there remains a critical lack of empirical research examining the law’s implications for those directly affected. Our study aims to address this gap by analyzing the lived experiences of individuals navigating the Austrian assisted suicide framework and evaluating its legal implementation. This approach is designed to contribute to the existing scholarship in evaluating assisted dying laws [17-21]. In addition to our scholarly objective, we aim to provide insights that may inform policy development and professional practice. To this end, we will disseminate our findings both within academia and beyond.

### Aims and Objectives

Our multiperspective evaluation aims to assess the newly enacted Dying Decree Act. By comparing and contrasting the perspectives of relevant key stakeholders, we aim to provide a nuanced understanding of the law’s societal, legal, and ethical implications while also exploring its practical consequences for the health care system and health care professionals. We define key stakeholders as individuals seeking assistance in dying; their family members and other close relatives; and all the professionals specified in the legal framework, notably physicians, notaries, other relevant legal professionals, pharmacists, and psychologists. Thus, our evaluation is grounded in a perspectivist approach, which maintains that any assessment of the law must be built upon the perspectives of those who are centrally affected by it, meaning the key stakeholders, as listed above [22,23]. Our study uses an interdisciplinary approach that incorporates theories, concepts, and methodologies from legal science, ethics, and social science.

### Research Questions

The research questions are as follows:

- What ethical and philosophical challenges arise from the implementation of the Dying Decree Act?
- How do key stakeholders experience and evaluate the law?
- What legal and practical issues arise from the implementation of the Dying Decree Act?

## Methods

### Legal Study Section

The study sets out to examine the Austrian legal framework for assisted suicide applying generally recognized methods of legal interpretation [24,25]. This analysis will be complemented by a comprehensive legal literature review of academic law journals, databases, and library resources. The review will focus on legal texts and their scholarly interpretation and dissemination, excluding empirical studies. Our aim lies in generating a structured understanding of the legal framework governing assisted suicide in Austria. Extracted material will be organized thematically according to key legal topics (eg, eligibility, procedural safeguards, and institutional responsibilities) to provide a structured narrative of the legal landscape. Searches will be conducted using relevant keywords related to the applicable statutory provisions.

Beyond the Austrian context, the study will also explore alternative regulatory approaches. This includes an analysis of other national and European Union (EU) provisions, guidelines, rulings, and proposed solutions. Particular attention will be paid to the case law of the European Court of Human Rights and further relevant EU documents as a means of illustrating the European framework in which the discourse on assisted suicide is embedded. In addition to EU regulations, the study will include a comparative legal analysis of how other nations regulate assisted suicide. By comparing these approaches with the Austrian framework, the study aims to provide both an overview of international best practice models and an insight into the Austrian legislators’ legal reasoning. The objective is twofold: (1) to understand the reasoning behind the Austrian legislators’ choices and (2) to apply a comparative perspective that provides the scientific community with new insights.

The legal analysis will be conducted by the authors in collaboration with legal experts from the Forum on End-of-Life Autonomy (Forum Autonomie am Lebensende). This forum is an expert panel that was established in 2022 and is headed by the Department for Ethics and Law in Medicine of the University of Vienna (Institut für Ethik und Recht in der Medizin). The forum members meet on a regular basis to discuss the practical implementation of the legal framework surrounding assisted suicide. It comprises experts from various fields with practical insights into the law and its application. By involving the forum, the study ensures that legal questions will be addressed and answered from multiple perspectives and areas of expertise.

### Ethics Study Section

The primary objective of this section of the study is to evaluate the moral implications and ethical challenges of the Dying Decree Act from a philosophical and applied ethical perspective. This is achieved by analyzing the line of argumentation used in the process of legalizing assisted suicide and its manifestation in the legal framework in Austria. We will then consider whether the Dying Decree Act can adequately address ethical concerns at the level of the individual, the health care system, and society as a whole.

Thus, we base our ethical reflections on a multiperspective, systemic approach. At the micro level, we focus on patient–health care professional interactions. Our applied ethics are guided by the 4 bioethical principles [26] as well as interprofessional health care ethics, referencing nursing and medical ethics as professional ethics. At the meso level, we focus on interactions between decision-makers and teams in health care settings, applying organizational ethics theories [27] from a systemic perspective. These highlight the urgent need for team and organizational development to implement the Dying Decree Act. At the macro level, it is important to clarify the various moral arguments influencing social discourse and reflect on managing tensions and conflicts in the Austrian health care system. This necessitates the active involvement of health care professionals, policymakers, and patients, ensuring that diverse perspectives are represented. It also demands careful attention to and respect for pluralism within a solidarity-based health care system. Therefore, the ethical analysis will consider practical consequences at the level of patients, health care professionals, and the health care system as a whole.

Building on this framework, we will select philosophical and ethical approaches equipped to identify and reflect on potential moral concerns associated with assisted suicide. Our aim is to show how these concerns and objections are more or less meaningful according to whether they target individuals, organizations, or society. Second, we will highlight those arguments and objections that arise during the legalization process and are specifically considered in the Dying Decree Act to discuss their consequences and implications for individuals, organizations, and society. We will focus primarily on the Austrian Constitutional Court texts as well as the legal text of the Dying Decree Act itself. Third, our ethical reflection is integrated into both the quantitative and qualitative research components.

### Social Science Study Section

#### Surveys

To date, the perspectives of physicians and those in leadership roles, such as medical and nursing directors, on this subject remain largely unexplored [28]. To address this gap, 2 online questionnaires were developed. One survey targets physicians of all specialties, whereas the other is targeted at directors of hospitals and long-term care facilities. By engaging both groups, the questionnaires provide valuable insights into their experiences with assisted suicide cases in Austria. Additionally, the surveys aim to determine whether physicians and directors of hospitals and nursing homes feel adequately supported in navigating the complex legal framework or whether additional resources and guidance are needed.

Findings from cross-sectional studies on assisted suicide conducted in various countries informed the development of the questionnaires [29-32]. In addition, experts in medical law, ethics, social science, public health, psychology, and medicine contributed to refining the questions and provided further input on the questionnaires’ structure and content. Before distribution, the questionnaires were pretested by the researchers regarding technical functionality.

We obtained a list of all hospitals and nursing facilities in Austria from the Ministry of Health and sent the questionnaires to each institution via email. For the physicians’ questionnaire, we asked the Austrian Medical Chamber to distribute the questionnaire to all practicing physicians in Austria.

Both surveys consisted of a mix of closed- and open-ended questions (24 items in the medical and nursing directors’ survey and 33 items in the physicians’ survey). The surveys commenced with sociodemographic questions regarding age, gender, and length of employment at their respective institutions. The main part of the questionnaire can thematically be grouped into (1) knowledge and understanding of the Dying Decree Act, (2) encounters and experiences with assisted suicide, (3) institutional guidelines, and (4) participants’ reported need for more information and support regarding assisted suicide. Considering the sensitive nature of the study, participants were allowed to skip questions.

Both questionnaires were programmed using the online tool SoSci Survey (version 3.5.02; SoSci Survey GmbH) [33]. The Cochran formula was used to calculate the required sample size by incorporating the estimated size of the target population as well as the desired α error and confidence coefficient. An α error of 5% was selected for both questionnaires, corresponding to a confidence coefficient of 95%. As there were 50,631 registered physicians in Austria in 2023 [34], the calculation indicated that a total of 385 participants would be required to obtain a representative sample to gain a first insight into Austrian physicians’ experiences and perceived challenges regarding assisted suicide. For the questionnaire targeted at medical and nursing directors, our population of interest consisted of 1251 Austrian health facilities, and we calculated that a minimum of 295 completed surveys would be needed.

We used purposive sampling by distributing the questionnaire targeted at physicians directly via the Austrian federal and state medical boards to reach our target population of physicians residing in Austria. Publicly available catalogs maintained by governmental institutions listing all nursing and medical facilities in Austria were used to directly contact the medical and nursing directors’ respective facilities.

For the data analysis, we started by conducting a descriptive statistical analysis of our sample using bar charts and contingency tables. This step focused on examining the distribution of responses to each item in the questionnaires to gain an initial overview of participants’ answers to our survey. On the basis of the identified patterns in the descriptive results, we subsequently conducted exploratory inferential analyses to further investigate potential associated variables within our data. As the data derived from the questionnaires primarily consisted of dichotomous (yes or no) variables and categorical variables, which correspond to a nominal scale level, participants’ answers were mainly analyzed using nonparametric methods, such as chi-square tests. ANOVAs and 2-tailed *t* tests were used to investigate differences in relation to the few metrically scaled items contained in the questionnaire (eg, Likert scales).

An English translation of the complete surveys can be found in the designated Open Science Framework (OSF) repository [35]. The CHERRIES (Checklist for Reporting Results of Internet E-Surveys) checklist was used to guide the reporting of the survey [36] as provided in Checklist 1.

#### Semistructured Interviews

We are conducting semistructured in-depth interviews with (1) individuals seeking assistance in dying; (2) family members and other relatives of individuals seeking assistance in dying; and (3) professionals mentioned in the legal framework, notably physicians, notaries and other relevant legal professionals, pharmacists, and psychologists. Interviews are conducted either online via a videoconferencing platform or in person at a location chosen by the participants (eg, their workplace or home). All interviews are audio recorded using the recording function of the corresponding videoconferencing platform or a digital voice recorder and subsequently transcribed verbatim by a professional transcriber. In addition, field notes are taken to document contextual details and supplement the content of each interview.

We recruit participants nationwide across all stakeholder groups to identify potential regional differences (Table 1). Calculations about the number of interviews and when to stop data collection cannot be determined in advance of analysis as such calculations are not consistent with the interpretative epistemological foundation of our interview study. In an iterative process, we recruit participants in parallel with transcribing and analyzing interviews to best determine when saturation is reached. Saturation is evaluated separately for each interview sample group and defined as the point at which no new information, codes, or themes emerge [37]. This process, in turn, ensures a comprehensive and thorough understanding of the law’s effects.

**Table 1.** Recruitment procedures for the interview study.

	Professionals mentioned in the legal framework	Individuals (or their family members and other relatives) seeking assistance in dying
Inclusion criteria	Professionals working in Austria with relevant experience according to their legally defined role in the Dying Decree Act	Adults (aged ≥18 years) residing in Austria (or their adult relatives) currently seeking or planning on seeking assistance in dying
Recruitment strategy	Nationwide recruitment via official professional associations and the Forum on End-of-Life Autonomy, supplemented by snowball sampling	A call for participation will be disseminated through relevant organizations and professionals connected to the practice of assisted suicide in Austria; if interested, potential interview participants will be able to contact the research group via the contact information provided in the call for study participation

All interviews follow an interview guide developed by the research team. An outline of the interview guide is provided in Multimedia Appendix 1, whereas the full version can be found in the designated OSF repository [35]. Different versions of the interview guide were prepared and adapted for each key stakeholder, tailoring them to the particularities of the stakeholders’ specific roles. However, the different versions of the interview guide are similar in terms of the central questions on the interviewees’ personal attitudes toward assisted suicide, their experiences with assisted suicide in Austria and potentially other countries, and their opinion on the Austrian law. Throughout the course of the study, the interview guides are continuously revised, expanded, and adapted. Interviews are prepared to accommodate unplanned questions that arise during the course of the dialogue. All interviewers have training and experience in leading interviews prior to the study and are familiar with the sensitive nature of the research topic. Furthermore, most interviews are conducted in pairs—with one lead interviewer and a notetaker also tasked with ensuring that all aspects of the interview guide are addressed.

We are conducting a thematic analysis to interpret and make sense of the interview data following the approach outlined by Braun and Clarke [38,39] and using the MAXQDA software (VERBI GmbH) [40]. This method facilitates comprehensive understanding of the dataset by identifying and analyzing latent patterns that encapsulate the overarching narrative conveyed by the data. Thematic analysis provides a framework for uncovering meaningful insights by systematically exploring how various aspects of the data interconnect. The process of analysis begins with an in-depth familiarization with the data, achieved through the repeated and immersive reading of transcribed interviews. To develop themes, we systematically search the interview transcripts for recurring passages and shared ideas, identifying clusters of meaning that reflect common messages or concepts. These passages are then compared and collated, enabling preliminary themes to be articulated. The next phase is to review and refine these themes by consistently relating them back to the original interview data, ensuring that they remain grounded in the participants’ perspectives and experiences. To strengthen interpretative validity and reduce potential researcher bias, the analysis is conducted collaboratively by multiple members of the research team. Furthermore, the researchers debrief on a regular basis in structured team discussions as a reflexivity practice [41].

Final reporting of the qualitative component of the study will be in accordance with the COREQ (Consolidated Criteria for Reporting Qualitative Research) guidelines as provided in Checklist 2, which ensures transparent and comprehensive reporting of the study design, data collection, and analysis [42].

### Ethical Considerations

This study is designed to comply with the Declaration of Helsinki. The University of Vienna Ethics Committee approved the initial research proposal on November 27, 2023 (01071). An addendum was submitted in September 2024, to which ethical consent was granted on November 4, 2024.

All participants were asked to provide informed consent prior to taking part. For the online survey, participants provided informed consent by clicking a confirmation button prior to proceeding to the questionnaire. Before being asked to provide their consent, participants were presented with detailed information about the study, including its purpose, the voluntary nature of participation, and data protection measures. The survey was conducted anonymously. Providing contact details for potential participation in a follow-up interview was optional, and they were collected separately from the survey responses.

For the interview study, all participants are asked to provide written informed consent using the official consent form issued by the Ethics Committee of the University of Vienna. Participation is voluntary, and participants may withdraw from the study at any time without providing a reason. Due to the sensitive nature of the topic, interview participants who are either individuals seeking assistance in dying or family members or relatives will also be informed that professional counseling services are available and may be accessed through the study team if desired.

No financial compensation or incentives were offered for participation.

Furthermore, all applicable data protection regulations, including the EU’s General Data Protection Regulation, have been strictly adhered to, and concise rules on data security have been formulated (eg, all data will be deleted when the research is concluded and results are published). All interviews will be transcribed by a professional transcriber who is subject to strict confidentiality requirements. All identifying details (eg, names and places) will be removed after transcription. Transcripts will be coded using unique identification numbers in accordance with pseudonymization procedures, and identifying information will be stored separately in a password-protected file within the secure university data system until completion of the project.

## Results

The study was funded in November 2023. The literature review and data collection for the legal and ethical analyses commenced in January 2024 and will continue until the study’s completion. Recruitment and data collection for the interview study began in January 2024 and are expected to be completed by April 2026. In total, 49 participants have been interviewed as of February 2026, including 5 (10.2%) individuals seeking assistance in dying, 10 (20.4%) family members and other relatives of individuals seeking assistance in dying, and 34 (69.4%) professionals mentioned in the legal framework (with physicians making up the largest demographic group with n=16, 47.1% interviewees). One online survey was launched in June 2024, the other in August 2024. The final samples consisted of 239 medical and nursing directors and 304 physicians. Data collection ended in October 2024, whereas data analysis was completed in May 2025. Completion and dissemination of all study components are anticipated by the end of 2026. Given the novelty of the legislation and the limited practical experience to date, further interviews are scheduled until April 2026.

## Discussion

### Expected Contribution

Our study aims to provide a comprehensive understanding of the lived experiences and practical challenges encountered by all stakeholders involved in assisted suicide under Austria’s newly established legal framework. By examining these perspectives, the study seeks to address critical gaps in empirical research and inform future policy and practice. Previous scholarship has highlighted that end-of-life regulation cannot be adequately understood through isolated legal or ethical analysis alone but requires a holistic approach [43]. In line with this identified need, our study uses an interdisciplinary approach to evaluate Austria’s new framework on assisted suicide, bringing stakeholders’ perspectives and lived experiences to the forefront. The study components, which incorporate multiple stakeholder perspectives, will aim to advance academic discourse by examining the experiences of (1) individuals seeking assistance in dying, (2) their family members and other relatives, and (3) involved professionals. Thus, this study generates empirical insights into how the legal framework shapes clinical roles, responsibilities, and accessibility. Ultimately, the study aims to bridge the gap between theoretical discussions on assisted suicide and the practical realities faced by those involved by offering evidence-based recommendations that are relevant not only to Austria but also to international debates on assisted dying legislation.

### Potential Findings and Comparison With Existing Literature

With our project, we aim to contribute to the international landscape of research on assisted dying regulation. It is essential to recognize that cultural and legal differences necessitate caution when transferring findings across countries. It is crucial to consider the lived experiences of all involved parties within their specific national context. An example illustrating this need is the variation in physicians’ attitudes and practices regarding assisted dying across countries [44-46].

In other countries, studies have already adopted multiperspective and interdisciplinary approaches to gain a comprehensive understanding of national practices and their impact on all involved stakeholders [18,43]. In Austria, research on assisted suicide has largely been limited to specific stakeholder groups [47-50], leaving a gap in understanding the interconnected nature of these groups’ various experiences. Our study is the first holistic approach evaluating the Austrian legal framework.

We anticipate that the framework’s lack of guidance regarding the final steps—where health care providers are neither required nor formally instructed to accompany individuals—creates practical uncertainties across multiple stakeholder groups. This may shape the experiences of individuals seeking assisted dying, their relatives, and health care professionals by influencing access, redistributing responsibilities, and creating uncertainty regarding professional roles. Health care institutions, including hospitals and nursing homes, may also encounter procedural ambiguities due to the informal handling of assisted suicide.

Additional anticipated findings include physicians’ reasoning regarding participation in the assessment process and eligibility determination, the (emotional) impact of assisted dying on relatives, and practical or financial barriers influencing equitable access to assisted suicide.

### Strengths and Limitations

The study sets out to provide a robust and practical study design capable of providing a thorough evaluation of the newly enacted law on assisted suicide from a multiperspective standpoint. Its large-scale approach is enabled by an interdisciplinary research team and a clear emphasis on including all stakeholder groups affected by the legal framework. This inclusive perspective—combined with a mixed methods approach suitable for collecting both qualitative and quantitative data—positions the study as the most extensive research design on this topic in Austria. As such, it is able to make a significant contribution to the research in this highly relevant field.

We are aware that our evaluation will face several challenges that may turn into limitations of our study. First, the recent enactment of the law may lead to a lack of routine in its practical implementation, which could cause the relevance of some identified issues to shift over time. Second, given the highly sensitive and personal nature of assisted dying, recruiting study participants—particularly individuals seeking assisted suicide and their family members—may prove challenging. Third, we need to take care of avoiding response bias. Study participants may be hesitant to fully disclose their experiences or opinions due to fear of judgment or perceived legal and professional repercussions. We aim to mitigate this by using state-of-the-art interviewing techniques and ensuring strict confidentiality throughout the research process.

### Dissemination

The results of this study will be disseminated to a broad audience through a variety of publications and international and national conferences. To maximize accessibility and facilitate unrestricted knowledge exchange, the results will be submitted to journals that support open-access publication. Additionally, the study has been preregistered through the OSF repository [35]. For the general public, the findings will be presented in plain, accessible language on the designated website. Finally, study results will be presented at roundtable discussions and expert panels organized by the Department of Ethics and Law in Medicine to ensure public engagement and knowledge exchange until the final research findings can be presented at the institute’s closing conference.

## Supplementary material

10.2196/86740 Multimedia Appendix 1 Interview guide outline.

10.2196/86740 Checklist 1 CHERRIES checklist.

10.2196/86740 Checklist 2 COREQ checklist.

## Abbreviations

CHERRIES: Checklist for Reporting Results of Internet E-Surveys
COREQ: Consolidated Criteria for Reporting Qualitative Research
EU: European Union
OSF: Open Science Framework
