# Supplementary material for: Evaluating Experiences With the Newly Enacted Law on Assisted Suicide in Austria: Protocol for an Interdisciplinary Mixed Methods Study
Source: JMIR Res Protoc. 2026 Apr 16;15:e86740. doi: 10.2196/86740 (PMC13086061; doi:10.2196/86740)
Supplement: Multimedia Appendix 1 [file resprot-v15-e86740-s001.pdf]

Outline Interviewguides

„Evaluating experiences of the newly enacted law on assisted suicide in Austria: protocol for an interdisciplinary mixed methods study"

Full versions may be obtained from corresponding author and from OSF after publication of the designated study part.

Individuals seeking assisted suicide

- Explanation from the interviewer to the participant about the research, its purpose, and informed consent
- Introduction of participants and collection of demographic data
- Scene setting: Illness trajectory and development of the wish to die
  - Onset, triggers, and influencing factors
  - Particularly intense phases of the wish to die
  - Consideration of assisted suicide and decision-making process
  - Exploration of alternative support options (e.g., palliative, psychological, hospice care)
- The path to the law – perspective on legal assisted suicide in Austria
  - Reaction to the Constitutional Court decision
  - Consideration of assisted suicide before legalization (including abroad)
  - Assessment of regulations in Austria compared to other countries
- Content of the law and associated challenges
  - Views on the Dying Decree Law and its implementation
  - Perceived adequacy of legal requirements
  - Practical steps toward obtaining a Dying Decree
- Assisted suicide in practice – wishes and preferences
  - Preferred circumstances, place, and presence of others
  - Role of family, friends, and medical personnel
- Information, counseling, and support
  - Access to information and first points of contact
  - Perceived sufficiency of guidance and involvement
- Retrospective: Key experiences and particularly significant aspects of the process
- Outlook and wishes for the future
  - Recommendations to legislators and stakeholders
  - Additional comments
- Closing: Information about next study steps, contact details, and thanks

Family members and other relatives of individuals seeking assisted suicide

- Explanation from the interviewer to the participant about the research, its purpose, and informed consent

- Introduction of participants and collection of demographic data

- Scene setting: Illness and development of the wish to die
    - How the wish to die was communicated and handled
    - Decision-making process for assisted suicide

- Options on the way to the end of life
    - Consideration of alternatives (e.g., assisted suicide abroad, Advance Care Planning)
    - Involvement of family or caregivers in exploring options

- The path to death – process and experiences
    - Steps from the wish to die to creation of the Dying Decree and medication
    - Support and challenges in creating the Dying Decree
    - Obtaining the medication (if applicable)
    - Experience of the day of death and implementation of assisted suicide
    - Involvement in planning and execution (presence, support, decision-making)
    - Estate and post-death arrangements

- Support and information for caregivers
    - Availability and accessibility of professional support
    - First points of contact and institutional support
    - Communication by authorities and adequacy of guidance

- Retrospective: Key experiences and significant aspects of the process

- Outlook and wishes for the future
    - Recommendations to legislators and stakeholders
    - Additional comments

- Closing: Next steps in the study, contact information, and thanks

Professions specified in the legal framework

- Explanation from the interviewer to the participant about the research, its purpose, and informed consent

- Introduction of participants and collection of demographic data

- Scene setting: Reason for participating in the study and participants' first insights into the topic
- The path to the law – expectations and initial considerations regarding implementation (with regard to professional group and personal situation)
    - Reactions within the professional group to the Constitutional Court ruling

- Assessment of the Court’s request to revise the full criminalization of assisted suicide
- Wishes and expectations regarding the legislative implementation prior to presentation of the law

- Content of the law and associated challenges
    - General assessment of the Dying Decree Law (StVfG)
    - New or potential responsibilities arising from the law
    - Evaluation of the law in relation to professional duties

- Practical impact and implementation
    - Encounters with the law since its entry into force
    - Forms of involvement in practice
    - Challenges in implementing the law within the professional group
    - Necessary framework conditions for effective implementation

- Information, training, and professional involvement
    - Involvement in the legislative process
    - Availability and extent of training or informational materials
    - Need for further education or clarification
    - Independent initiatives (e.g., development of materials or training offers)
    - Evaluation of communication by responsible authorities

- Outlook and wishes for the future
    - Recommendations to legislators
    - Additional comments

- Closing: Next steps in the study, contact information, and thanks
