# Supplementary material for: Evaluating Experiences With the Newly Enacted Law on Assisted Suicide in Austria: Protocol for an Interdisciplinary Mixed Methods Study
Source: JMIR Res Protoc. 2026 Apr 16;15:e86740. doi: 10.2196/86740 (PMC13086061; doi:10.2196/86740)
Supplement: Checklist 1 [file resprot-v15-e86740-s002.pdf]

Checklist for Reporting Results of Internet E-Surveys (CHERRIES)

Checklist Item	***Explanation***	***Page Number***
Describe survey design	Describe target population, sample frame. Is the sample a convenience sample? (In "open" surveys this is most likely.)	The target population consisted of physicians of all specialties as well as managers of hospitals and long-term care facilities. Purposive sampling was used.
IRB approval	Mention whether the study has been approved by an IRB.	Yes, the study has been approved by the University of Vienna Ethics Committee in November 2023.
Informed consent	Describe the informed consent process. Where were the participants told the length of time of the survey, which data were stored and where and for how long, who the investigator was, and the purpose of the study?	Participants' consent was obtained by clicking on the "continue"-button on the first page of the questionnaire. The first page also contained information about length, purpose and data use as well as the investigators' name and contact information.
Data protection	If any personal information was collected or stored, describe what mechanisms were used to protect unauthorized access.	Data was stored on a secure and encrypted university server.
Development and testing	State how the survey was developed, including whether the usability and technical functionality of the electronic questionnaire had been tested before fielding the questionnaire.	We conducted a pretest to account for technical issues with the questionnaire before fielding the survey.
Open survey versus closed survey	An "open survey" is a survey open for each visitor of a site, while a closed survey is only open to a sample which the investigator knows (password-protected survey).	The survey wasn't password-protected; however, access was only possible via a specific link that was sent to the selected population.
Contact mode	Indicate whether or not the initial contact with the potential participants was made on the Internet. (Investigators may also send out questionnaires by mail and allow for Web-based data entry.)	The questionnaires were distributed via email and data was collected and managed using the survey platform SoSciSurvey.com (Version 3.5.02).
Advertising the survey	How/where was the survey announced or advertised? Some examples are offline media (newspapers), or online (mailing lists – If yes, which ones?) or banner ads (Where were these banner ads posted and what did they look like?). It is important to know the wording of the announcement as it will heavily influence who chooses to participate. Ideally the survey announcement should be published as an appendix.	The surveys were neither publicly announced nor advertised; the link was sent directly to hospital/care facility managers of selected institutions that met our inclusion criteria (e.g. offer palliative care, geriatric or long-term care services that treat adult patients). The survey concerning physicians was sent out via the Austrian Medical

		Chamber, where all practicing physicians are registered.
Web/E-mail	State the type of e-survey (eg, one posted on a Web site, or one sent out through e-mail). If it is an e-mail survey, were the responses entered manually into a database, or was there an automatic method for capturing responses?	It was an e-mail survey. Participants were led to SoSciSurvey via a link and the survey platform captured responses automatically.
Context	Describe the Web site (for mailing list/newsgroup) in which the survey was posted. What is the Web site about, who is visiting it, what are visitors normally looking for? Discuss to what degree the content of the Web site could pre-select the sample or influence the results. For example, a survey about vaccination on a anti-immunization Web site will have different results from a Web survey conducted on a government Web site	SoSciSurvey is an online platform that is specifically designed for creating and distributing online surveys. To ensure data security and privacy, we accessed SoSciSurvey via a hosted server of the University of Vienna.<br><br>As the link was only sent to individuals meeting our inclusion criteria, it was not expected that the survey website would influence the sample or the results.
Mandatory/voluntary	Was it a mandatory survey to be filled in by every visitor who wanted to enter the Web site, or was it a voluntary survey?	Survey participation was voluntary.
Incentives	Were any incentives offered (eg, monetary, prizes, or non-monetary incentives such as an offer to provide the survey results)?	No incentives were offered for participation in the survey.
Time/Date	In what timeframe were the data collected?	Data collection for both questionnaires took place from June to October 2024
Randomization of items or questionnaires	To prevent biases items can be randomized or alternated.	Item randomization was not applied as some of the questions built upon questions that were asked before and randomization would therefore have disrupted the logical flow of the questionnaire.
Adaptive questioning	Use adaptive questioning (certain items, or only conditionally displayed based on responses to other items) to reduce number and complexity of the questions.	Adaptive questioning was used to gain more detailed information in relation to certain answers (for example if a person responded to have had encountered a person with an established Dying Decree, they were led to a follow up question asking them **how many encounters** with persons with a Dying Decree they had experienced **exactly**)

Number of Items	What was the number of questionnaire items per page? The number of items is an important factor for the completion rate.	Total number of items in physicians' survey = **33**<br>Total number of items in hospital/care facility managers' survey = **24**<br><br>Items per page varied between 1 und 4 (except for sociodemographic page, which included 7 sociodemographic items)
Number of screens (pages)	Over how many pages was the questionnaire distributed? The number of items is an important factor for the completion rate.	Number of pages in physicians' survey = **20**<br>Number of pages in hospital/care facility managers' survey = **16**
Completeness check	It is technically possible to do consistency or completeness checks before the questionnaire is submitted. Was this done, and if "yes", how (usually JAVAScript)? An alternative is to check for completeness after the questionnaire has been submitted (and highlight mandatory items). If this has been done, it should be reported. All items should provide a non-response option such as "not applicable" or "rather not say", and selection of one response option should be enforced.	There were no completeness checks made before submission of the questionnaire. We checked for completeness after the questionnaire had been submitted, however, due to the highly sensitive topic of the questionnaire, none of the items were designed as mandatory items and participants were allowed to skip items.
Review step	State whether respondents were able to review and change their answers (eg, through a Back button or a Review step which displays a summary of the responses and asks the respondents if they are correct).	Participants were allowed to navigate freely through the questionnaire using a Back button.
Unique site visitor	If you provide view rates or participation rates, you need to define how you determined a unique visitor. There are different techniques available, based on IP addresses or cookies or both.	N.A.
View rate (Ratio of unique survey visitors/unique site visitors)	Requires counting unique visitors to the first page of the survey, divided by the number of unique site visitors (not page views!). It is not unusual to have view rates of less than 0.1 % if the survey is voluntary.	N.A.
Participation rate (Ratio of unique visitors who agreed to participate/unique first survey page visitors)	Count the unique number of people who filled in the first survey page (or agreed to participate, for example by checking a checkbox), divided by visitors who visit the first page of the survey (or the informed consents page, if present). This can also be called "recruitment" rate.	N.A.

Completion rate (Ratio of users who finished the survey/users who agreed to participate)	The number of people submitting the last questionnaire page, divided by the number of people who agreed to participate (or submitted the first survey page). This is only relevant if there is a separate "informed consent" page or if the survey goes over several pages. This is a measure for attrition. Note that "completion" can involve leaving questionnaire items blank. This is not a measure for how completely questionnaires were filled in. (If you need a measure for this, use the word "completeness rate".)	Physicians' survey:<br>completion rate = **79%** (305 out of 385 participants who filled out the first page of the questionnaire completed the whole survey)<br><br>Hospital/care facility managers' survey:<br>completion rate = **76%** (199 out of 262 participants who filled out the first page of the questionnaire completed the whole survey)
Cookies used	Indicate whether cookies were used to assign a unique user identifier to each client computer. If so, mention the page on which the cookie was set and read, and how long the cookie was valid. Were duplicate entries avoided by preventing users access to the survey twice; or were duplicate database entries having the same user ID eliminated before analysis? In the latter case, which entries were kept for analysis (eg, the first entry or the most recent)?	N.A.
IP check	Indicate whether the IP address of the client computer was used to identify potential duplicate entries from the same user. If so, mention the period of time for which no two entries from the same IP address were allowed (eg, 24 hours). Were duplicate entries avoided by preventing users with the same IP address access to the survey twice; or were duplicate database entries having the same IP address within a given period of time eliminated before analysis? If the latter, which entries were kept for analysis (eg, the first entry or the most recent)?	N.A.
Log file analysis	Indicate whether other techniques to analyze the log file for identification of multiple entries were used. If so, please describe.	No cookies, IP checks or other types of log file analysis were used as they can reveal identifiable information, and participant privacy was of highest importance due to the topic of the study.
Registration	In "closed" (non-open) surveys, users need to login first and it is easier to prevent duplicate entries from the same user. Describe how this was done. For example, was the survey never displayed a second time once the user had filled it in, or was the username stored together with the survey	N.A.

	results and later eliminated? If the latter, which entries were kept for analysis (eg, the first entry or the most recent)?	
Handling of incomplete questionnaires	Were only completed questionnaires analyzed? Were questionnaires which terminated early (where, for example, users did not go through all questionnaire pages) also analyzed?	Items from partially completed questionnaires were included in the analyses whenever responses were available, as we specifically permitted skipping answers due to the delicate subject. However, cases in which no items were answered after the sociodemographic section were excluded as they did not provide any relevant information for analysis.
Questionnaires submitted with an atypical timestamp	Some investigators may measure the time people needed to fill in a questionnaire and exclude questionnaires that were submitted too soon. Specify the timeframe that was used as a cut-off point, and describe how this point was determined.	Timeframes were analyzed and cases with suspiciously small timeframes (Relative Speed Index > 2) were excluded from further data analysis.
Statistical correction	Indicate whether any methods such as weighting of items or propensity scores have been used to adjust for the non-representative sample; if so, please describe the methods.	N.A.

This checklist has been modified from Eysenbach G. Improving the quality of Web surveys: the Checklist for Reporting Results of Internet E-Surveys (CHERRIES). J Med Internet Res. 2004 Sep 29;6(3):e34 [erratum in J Med Internet Res. 2012; 14(1): e8.]. Article available at https://www.jmir.org/2004/3/e34/; erratum available https://www.jmir.org/2012/1/e8/. Copyright ©Gunther Eysenbach. Originally published in the Journal of Medical Internet Research, 29.9.2004 and 04.01.2012.

This is an open-access article distributed under the terms of the Creative Commons Attribution License (https://creativecommons.org/licenses/by/2.0/), which permits unrestricted use, distribution, and reproduction in any medium, provided the original work, first published in the Journal of Medical Internet Research, is properly cited.
