# Supplementary material for: Evaluating Experiences With the Newly Enacted Law on Assisted Suicide in Austria: Protocol for an Interdisciplinary Mixed Methods Study
Source: JMIR Res Protoc. 2026 Apr 16;15:e86740. doi: 10.2196/86740 (PMC13086061; doi:10.2196/86740)
Supplement: Checklist 2 [file resprot-v15-e86740-s003.pdf]

Consolidated criteria for reporting qualitative studies (COREQ): 32-item checklist

No. Item	Guide questions/description	Reported on Page #
**Domain 1: Research team and reflexivity**		
Personal Characteristics		
1. Inter viewer/facilitator	Which author/s conducted the interview or focus group?	TLV: Individuals seeking assistance in dying<br>TLV: Family members and other relatives<br>KD, JF: Professionals mentioned in the framework
2. Credentials	What were the researcher's credentials? E.g. PhD, MD	KD: Mag.iur<br>TLV: MSc<br>JF: Mag, PhD
3. Occupation	What was their occupation at the time of the study?	KD: Researcher<br>TLV: Researcher<br>JF: Researcher
4. Gender	Was the researcher male or female?	KD: Female<br>TLV: Female<br>JF: Female
5. Experience and training	What experience or training did the researcher have?	KD: Relevant studies, additional courses in qualitative theory, multiple finished studies<br>TLV: Relevant studies, additional courses in qualitative theory, multiple finished studies<br>JF: Relevant studies, additional courses in qualitative theory, multiple finished studies
Relationship with participants		
6. Relationship established	Was a relationship established prior to study commencement?	No prior relationship established
7. Participant knowledge of the interviewer	What did the participants know about the researcher? e.g. personal goals, reasons for doing the research	Affiliation, study design (e.g. aim of the study)

8. Interviewer characteristics	What characteristics were reported about the interviewer/facilitator? e.g. bias, assumptions, reasons and interests in the research topic	No characteristics reported
Theoretical framework		
9. Methodological orientation and Theory	What methodological orientation was stated to underpin the study? e.g. grounded theory, discourse analysis, ethnography, phenomenology, content analysis	Thematic analysis by Braun and Clarke
Participant selection		
10. Sampling	How were participants selected? e.g. purposive, convenience, consecutive, snowball	Purposive, snowball
11. Method of approach	How were participants approached? e.g. face-to-face, telephone, mail, email	Email
12. Sample size	How many participants were in the study?	About 50 in total (ongoing)
13. Non-participation	How many people refused to participate or dropped out? Reasons?	Refusals were documented;<br>0 dropouts
Setting		
14. Setting of data collection	Where was the data collected? e.g. home, clinic, workplace	Interviews are conducted online or at preferred location of the interviewee
15. Presence of non-participants	Was anyone else present besides the participants and researchers?	No one else was present besides the researchers and participants
16. Description of sample	What are the important characteristics of the sample? e.g. demographic data, date	Involvement in the procedure, job description, location/place of residency
Data collection		
17. Interview guide	Were questions, prompts, guides provided by the authors? Was it pilot tested?	Yes, short versions of the interview guides are provided as appendixes, and the long versions are found in the OSF repository<br><br>The interview guide was not pilot tested

18. Repeat interviews	Were repeat interviews carried out? If yes, how many?	There was no repetition of interviews
19. Audio/visual recording	Did the research use audio or visual recording to collect the data?	Audio recording
20. Field notes	Were field notes made during and/or after the interview or focus group?	Yes, field notes were taken to gain contextual details
21. Duration	What was the duration of the interviews or focus group?	Between 60-120 minutes
22. Data saturation	Was data saturation discussed?	Yes
23. Transcripts returned	Were transcripts returned to participants for comment and/or correction?	Transcripts were not returned to participants
**Domain 3: analysis and findings**		
Data analysis		
24. Number of data coders	How many data coders coded the data?	Two per interview group
25. Description of the coding tree	Did authors provide a description of the coding tree?	Duly considered
26. Derivation of themes	Were themes identified in advance or derived from the data?	Derived from Data
27. Software	What software, if applicable, was used to manage the data?	MAXQDA
28. Participant checking	Did participants provide feedback on the findings?	Duly considered
Reporting		
29. Quotations presented	Were participant quotations presented to illustrate the themes/findings? Was each quotation identified? e.g. participant number	Duly considered
30. Data and findings consistent	Was there consistency between the data presented and the findings?	Duly considered
31. Clarity of major themes	Were major themes clearly presented in the findings?	Duly considered
32. Clarity of minor themes	Is there a description of diverse cases or discussion of minor themes?	Duly considered
